# Diastolic Dysfunction Is a Predictor of Poor Survival in Patients with Decompensated Cirrhosis

**DOI:** 10.1155/2021/5592376

**Published:** 2021-12-02

**Authors:** Manas Kumar Behera, Surendra Nath Swain, Manoj Kumar Sahu, Gaurav Kumar Behera, Debakanta Mishra, Jimmy Narayan, Ayaskant Singh, Shobhit Agarwal, Pradeep Kumar Mallick

**Affiliations:** ^1^Department of Gastroenterology, IMS and SUM Hospital, Bhubaneswar, India; ^2^Department of Medicine, IMS and SUM Hospital, Bhubaneswar, India; ^3^Department of Cardiology, IMS and SUM Hospital, Bhubaneswar, India

## Abstract

**Background:**

Left ventricular diastolic dysfunction (LVDD) appears to be the earliest cardiac disturbance in cirrhosis patients. There are many previous reports reporting the significance of severity of LVDD on the outcome of liver transplantation or TIPS insertion, a few Indian studies have addressed the role of LVDD on survival in decompensated cirrhosis. The objective of this study is to assess the effect of LVDD on the survival of decompensated cirrhotic patients.

**Methods:**

We prospectively evaluated 92 decompensated cirrhotic patients from April 2015 to March 2017 at IMS and SUM Hospital, Bhubaneswar, India. 2D echocardiography with tissue Doppler imaging was used to evaluate cardiac function, as per the American society of echocardiography guidelines. The primary endpoint was to evaluate the effect of LVDD on overall mortality.

**Results:**

Ninety-two decompensated cirrhotic patients were evaluated in this prospective cohort study. Twenty-eight out of 92 patients (30%) died due to liver-related complications after a follow-up of 24 months. The decompensated cirrhotic patients with MELD score ≥ 15 had a significantly higher *E*/*e*′ ratio (11.94 ± 4.24 vs. 8.74 ± 3.32, *p* < 0.001) suggesting severe LV dysfunction in advanced cirrhosis. Patients with *E*/*e*′ ratio > 10 had significantly higher MELD score and Child-Pugh score (19.88 ± 7.72 vs. 14.31 ± 5.83; 10.25 ± 1.74 vs. 9.02 ± 1.74, *p* < 0.01, respectively) as compared to the*E*/*e*′ ratio < 10 group. In Cox proportional hazard multivariate analysis, *E*/*e*′ ≥ 10 (HR 2.72, 95% CI 1.07-6.9, *p* = 0.03) and serum albumin (HR 0.32, 95% CI 0.14-0.7, *p* < 0.01) were found to be independent predictors of mortality in decompensated cirrhotic patients.

**Conclusion:**

: The presence of LVDD and low serum albumin were independent predictors of mortality in decompensated cirrhotic patients. Hence, LVDD is an indicator of advanced cirrhosis and mortality.

## 1. Introduction

Cirrhosis of liver is the terminal stage of chronic liver disease, which is characterized by an early asymptomatic compensated phase followed by a rapidly progressive decompensated phase of the disease. The gross decline in liver function in decompensated cirrhosis can lead to the development of life-threatening complications like ascites, hepatic encephalopathy, jaundice, and variceal bleeding [1–3]. Cirrhosis of liver is a hyperdynamic state characterized by increased cardiac output (CO) and decreased systemic vascular resistance (SVR) due to splanchnic arterial vasodilatation secondary to portal hypertension [4, 5].

As per the World Congress of Gastroenterology, cirrhotic cardiomyopathy is an entity defined as “chronic cardiac dysfunction in patients with cirrhosis characterized by impaired contractile responsiveness to stress and/or altered diastolic relaxation with electrophysiological abnormalities in the absence of other known cardiac disease.” [6, 7] Activation of renin-angiotensin-aldosterone system, changes in membrane fluidity leading to impairment of *β* adrenergic receptor function, and accumulation of nitric oxide play a major role in the pathogenesis of cardiac dysfunction in cirrhosis of the liver [8, 9].

Left ventricular diastolic dysfunction (LVDD) appears to be an early cardiac disturbance in patients of cirrhosis of the liver, and studies have shown that LVDD is more severe in decompensated cirrhotic patients than compensated patients [9]. LVDD is also found to be a good predictor of poor survival in advanced liver disease and type 1 HRS [9, 10]. Neither electrocardiogram nor CTP and MELD can predict LVDD in cirrhosis of the liver. Hence, echocardiography is a simple, noninvasive modality to predict LVDD in cirrhotic patients accurately. Very few Indian studies have assessed the prognostic role of LVDD in decompensated cirrhotic patients in Indian patients. Therefore, we conducted a prospective study to evaluate the frequency of LVDD and to assess the impact of LVDD on survival in patients of decompensated liver cirrhosis.

## 2. Materials and Methods

### 2.1. Patient

A total of 92 cirrhotic patients with an age range of 18 to 65 years attending the gastroenterology department of IMS and SUM hospital from April 2015 to March 2017 were included in the study, regardless of the etiology. Cirrhosis of the liver was diagnosed by clinical, laboratory, imaging, or histologic findings. All these cirrhotic patients were decompensated, defined as Child-Pugh score ≥ 7 and/or the presence of ascites, encephalopathy, and variceal bleeding [10]. Patients with age > 65 years (*n* = 21), history of diabetes mellitus (*n* = 17), arterial hypertension (*n* = 4), chronic cardiac disease (*n* = 3), pulmonary or renal disease (*n* = 7), hepatocellular carcinoma (*n* = 6), and recent (within 6 months) or active ethanol abuse (*n* = 13) were excluded from the study. Those cirrhotic patients who were on *β* blocker therapy for treatment of variceal bleeding undergo repeated sessions of variceal band ligation until the variceal eradication. Following variceal eradication, these patients were instructed to stop *β* blocker 7 days prior to the cardiac function evaluation, after taking consent from the patients [24]. Patients who had active infection, Grade III/IV encephalopathy, GI bleed, or tense ascites were included in the study after one month of recovery of these complications. All study subjects were counselled about the opportunity of being included in the study for cardiovascular assessment by a qualified gastroenterologist, and informed consent was taken from all the participants. Informed consent was taken from all the patients, and the study was approved by the ethical committee.

### 2.2. Demographic and Clinical Data

A detailed history and thorough clinical examination were done at the time of admission or outpatient visit. A complete blood count, liver function test, renal function test, coagulation parameters, and electrolytes were measured. All these patients were on a sodium-restricted diet and without diuretics for at least 4 days prior to obtaining blood reports. Model for end-stage liver disease (MELD) and Child-Pugh (CTP) scores were calculated using these blood reports.

### 2.3. Echocardiography Data

All the cirrhotic patients underwent two-dimensional echocardiography with tissue Doppler imaging (TDI) (Vivid E9; General Electric, Boston, MA, USA) by an experienced cardiologist according to the recommendations of the American Society of Echocardiography (ASE) [11]. Left ventricular ejection fraction (LVEF %) was calculated by the modified Simpson's rule, left atrium volume index (LAVI), peak early filling velocity (*E*), atrial filling velocity (*A*), calculated *E*/*A* ratio (*E*/*A*), deceleration time of the E wave (DT), early diastolic mitral inflow velocity/velocity of the septal and lateral sites (*e*′) were estimated, and *E*/*e*′ ratio (*E*/*e*′) was calculated. LVDD was defined and classified according to the recommendations of the ASE [12] as follows: grade 1 LVDD: *e*′ < 8 cm/sec, *E*/*A* ratio < 0.8, *E*/*e*′ ratio < 9, and DT > 200 ms; grade 2 LVDD: *e*′ < 8 cm/sec, *E*/*A* ratio 0.8–1.5, *E*/*e*′ ratio 9–15, and DT 160–200 ms; and grade 3 LVDD: *e*′ < 8 cm/sec, *E*/*A* ratio > 2, *E*/*e*′ ratio > 15, and DT < 160 ms.

### 2.4. Statistical Analysis

The quantitative data with normal distribution were reported as mean ± SD, and comparisons were made by Student's *t*-test. Nonparametric quantitative data were reported as median with range and compared by the Mann–Whitney test. The qualitative data were compared by the chi-square test. For the estimation of survival, Kaplan-Meier analysis was done, and probability curves were compared by the log-rank test. The predictors of survival were calculated by Cox's proportion hazard model. Those variables with *p* value < 0.05 in univariate analysis were subjected to multivariate analysis by Cox's proportion hazard model. All the statistical analyses were performed by using SPSS 20.0 software (SPSS, Inc., Chicago, IL). A *p* value less than 0.05 was considered statistically significant.

## 3. Results

### 3.1. Baseline Clinical Characteristics

Ninety-two decompensated cirrhotic patients were evaluated in this prospective cohort study. The baseline characteristics of the study population are depicted in Table 1. The mean age of patients was 53 ± 9.85 years. The majority of participants were male with male : female ratio of 3.18 : 1. The most common cause of cirrhosis in the study population was alcohol (33%) followed by HBV (14%). Of all patients, 55.5% of patients belonged to Child-Pugh class C,36.9% belonged to class B and rest 7.6% belonged to class A. The mean serum albumin, serum bilirubin, serum creatinine, and INR were 2.61 ± 0.68 g/dl, 2.43 ± 1.85 mg/dl, 1.04 ± 0.63 mg/dl, and 2.43 ± 1.85, respectively. The mean MELD score of the study population was 16.73 ± 7.2, with a range of 7-36. The echocardiographic findings revealed that the mean LVEF, mitral *E*/*A* ratio, *E*/*e*′ ratio, DT, and LAVI were 61.76 ± 8.67%, 1.14 ± 0.46, 9.46 ± 3.24, 198.98 ± 32.33 ms, and 28.74 ± 9.42 ml/m^2^, respectively.

### 3.2. LVDD: Relation with Clinical and Echocardiographic Data

The clinical and echocardiographic data were classified according to *E*/*e*′ ratio and shown in Table 2. Diastolic dysfunction was classified on the basis of the *E*/*e*′ ratio (cut-off value: 10) into two groups as per recent guidelines [12]. Patients with *E*/*e*′ ratio > 10 had significantly higher MELD score, INR, and Child-Pugh score (19.88 ± 7.72 vs. 14.31 ± 5.83; 1.99 ± 0.65 vs. 1.46 ± 0.42; 10.25 ± 1.74 vs. 9.02 ± 1.74, *p* < 0.01, respectively) as compared to the *E*/*e*′ ratio < 10 group. The echocardiographic parameters like *E*/*A* ratio, *E*/*e*′ ratio, and LAVI were higher significantly in the *E*/*e*′ ratio > 10 group (1.34 ± 0.53 vs. 0.98 ± 0.32, *p* < 0.001; 12.67 ± 2.35 vs. 6.99 ± 0.55, *p* < 0.001; and 35.48 ± 6.73 vs. 24.08 ± 3.94, *p* < 0.001, respectively, and DT was significantly lower in the *E*/*e*′ ratio > 10 group (174.7 ± 21.02 vs. 217.65 ± 26.64, *p* < 0.001) as compared to the *E*/*e*′ ratio < 10 group. LV ejection fraction and mean age were found to be similar in both the groups.

### 3.3. LVDD: Classified by MELD Status

The echocardiographic data were classified as per MELD status and depicted in Table 3. The cirrhotic patients were classified on the basis of MELD score into two groups (MELD < 15 and MELD ≥ 15 groups) as MELD score > 15 was taken as the reference point for classifying CLD patients in a previous study [13]. The decompensated cirrhotic patients with MELD score ≥ 15 had significantly higher mitral *E*/*A* ratio, *E*/*e*′ ratio, and LAVI (1.31 + 0.69 vs. 1.01 ± 0.45, *p* < 0.01; 11.94 ± 4.24 vs. 8.74 ± 3.32, *p* < 0.001 and 33.11 ± 8.51 vs. 26.58 ± 6.23, *p* < 0.001, respectively). The DT was significantly lower in the MELD score ≥ 15 group than MELD < 15 group (185.45 ± 37.74 vs. 209.10 ± 33.20, *p* < 0.001) suggesting severe LVDD in patients with the MELD ≥ 15 group. Grade 2 LVDD was significantly more in patients with MELD ≥ 15 than the other group (38% vs. 23%). There was no difference in systolic function as estimated by LVEF in both groups.

### 3.4. LVDD: Relation with HRS Development

In our study, 15 patients (16%) developed hepatorenal syndrome, defined as per revised consensus recommendations of the International Club of Ascites 2015 [14]. Fourteen patients (15%) developed hepatic encephalopathy, 13 patients (14%) developed variceal bleeding, and 21 patients (23%) developed infectious complications like spontaneous bacterial peritonitis, urinary tract infection, and pneumonia requiring hospitalization. Decompensated cirrhotic patients with moderate ascites (*n* = 63) who developed HRS were compared with those did not develop HRS during follow-up, presented in Table 4. Patients developing HRS showed higher MELD score (24.92 ± 9.35 vs. 14.56 ± 5.43, *p* < 0.01), Child-Pugh score (10.92 ± 1.49 vs. 9.38 ± 1.62, p <0.01), *E*/*e*′ ratio (12.06 ± 3.22 vs. 8.86 ± 2.73, *p* < 0.01) and LAVI (34.21 ± 9.06 vs. 28.51 ± 7.51, *p* < 0.05) as compared to patients did not develop HRS. The prevalence of grade 2 LVDD was higher with patients who developed HRS than those who did not develop HRS (67.7% vs. 39.5%, *p* < 0.01). No significant differences in systolic function were observed between both groups.

### 3.5. Predictive Factors for Survival in DCLD Patients

**Figure 1:**
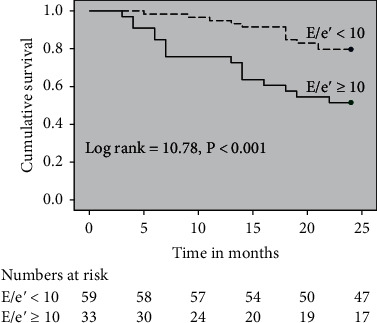
Kaplan-Meier survival curve of decompensated cirrhosis patients according to *E*/*e*′ values. Those patients having *E*/*e*′ ≥ 10 had lower survival rate than patients than *E*/*e*′ < 10 (*p* = 0.001).

At the end of a 24-month follow-up, 28 out of 92 patients (30%) died due to liver-related complications. Out of 28 patients,16 patients died in the *E*/*e*′ ratio > 10 group (16/33, 48%) and 12 patients died in the *E*/*e*′ ratio < 10 group (12/59, 20%). In Kaplan-Meier survival analysis, patients with *E*/*e*′ ratio > 10 group had significantly lower survival than the patients with the *E*/*e*′ ratio < 10 group (17.30 ± 1.32 vs. 22.55 ± 0.85, *p* < 0.01) with a log-rank value of 10.44 as shown in Figure 1. The causes of death were due to sepsis (*n* = 6), hepatorenal syndrome (*n* = 6), hepatic failure (*n* = 5), variceal bleeding (*n* = 5), and multiorgan failure (*n* = 6).

The factors predictive of mortality in decompensated cirrhotic patients are shown in Table 5. Age, male sex, serum albumin, MELD score, presence of ascites, alcohol vs. other etiology, LVEF, and *E*/*e*′ ratio were analysed by the Cox proportion hazard model. In univariate analysis, serum albumin (HR = 0.11, 95% CI 0.05-0.33, *p* < 0.001), MELD score (HR = 1.05, 95% CI 1.01-1.16, *p* < 0.01),Child-Pugh score (HR = 1.52, 95% CI 1.2-1.94, *p* < 0.01), presence of ascites (HR = 2.02, 95% CI 1.13-4.16,*p* < 0.05), and *E*/*e*′ ≥ 10 (HR = 4.19, 95% CI 1.78-9.88, *p* < 0.001) were found to be significant predictors of mortality. However, in multivariate analysis, *E*/*e*′ ≥ 10 (*HR* = 2.72, 95% CI 1.07-6.9, *p* = 0.03) and serum albumin (HR = 0.32, 95% CI 0.14-0.7, *p* < 0.01) were found to be independent predictors of mortality in decompensated cirrhosis patients. The presence of ascites was not a significant predictor of mortality in multivariate analysis (*p* = 0.48).

## 4. Discussion

Cirrhotic cardiomyopathy is considered hemodynamic alteration in patients with cirrhosis of liver, resulting in decreased systemic vascular resistance and increased cardiac output, causing excessive cardiac workload [15, 16]. Studies had shown that cirrhotic cardiomyopathy is associated with worsening of course of disease in cirrhosis of liver, like hepatorenal syndrome after spontaneous bacterial peritonitis, and decreased survival after implantation of transjugular intrahepatic portosystemic shunts [17, 18].

The prevalence of LVDD in our study was 66.3%, relatively higher as compared to the previously reported rate of 50% in cirrhotic patients [19]. A prospective study by Lee et al. from Korea reported an LVDD prevalence rate of 62.7% in cirrhotic patients, as similar to the findings of our study [10]. Our study population was advanced cirrhotic patients with more severe cardiac and circulatory dysfunction; hence, we reported a higher rate of LVDD as compared to previous studies. Systolic functions, measured by LVEF, were similar in cirrhotic patients with the *E*/*e*′ ratio ≥ 10 group and *E*/*e*′ ratio < 10 group. Most of the previous studies had similar observations that diastolic dysfunction precedes clinical heart failure in cirrhotic patients, as reported in our study [10, 20, 21]. Even though systolic function at rest was normal in cirrhosis of liver, these patients are prone to develop cardiac failure under stressful conditions, due to the presence of underrecognized subclinical LV diastolic dysfunction [22, 23]. Echocardiographic parameters *E*/*A* ratio, *E*/*e*′ ratio, and deceleration time were the factors used to measure diastolic dysfunction in previous studies. However, recent guidelines have suggested that the *E*/*e*′ ratio is an important modality to diagnose and grade LVDD as per the recent guidelines. Higher *E*/*e*′ ratio (>10) is associated with higher grades of diastolic dysfunction [10, 12].

In our study, the decompensated cirrhotic patients with *E*/*e*′ ratio ≥ 10 had significantly higher Child-Pugh score, MELD score, and lower serum albumin as compared to patients with *E*/*e*′ ratio < 10.This association of *E*/*e*′ ratio with the severity of liver disease is also found in previous studies. A prospective study from Spain by Ruiz del Arbol et al. established LVDD as the sensitive marker of advanced cirrhosis and mortality, with the shortest probability of survival found in cirrhotic patients with grade 2 LVDD [24]. Another study from Greece found to have a positive correlation of Child-Pugh score and a negative correlation of serum albumin with the severity of LVDD [25]. A Korean prospective study of 70 decompensated cirrhotic patients reported a significantly lower survival in the LVDD group as compared to patients without LVDD [10]. Hence, the deterioration of cardiocirculatory function in advanced cirrhosis could be the cause attributable to higher mortality in patients of cirrhosis with LVDD.

In our study, cardiac diastolic parameters were significantly different in cirrhotic patients with MELD ≥ 15 than MELD < 15, suggesting a higher degree of diastolic dysfunction with advanced cirrhosis. Multiple studies had evaluated the role of MELD status with cardiac function in cirrhosis of liver. A systematic review by Stundiene et al. demonstrated a significant correlation of MELD score and diastolic dysfunction with substantial difference in means of MELD score between grade 0 vs. grade 3 LVDD and grade 1 and grade 3 LVDD [26]. A previous study by Anish et al. also found a significantly higher MELD score in grade 2 LVDD as compared to grade 1 LVDD and no LVDD [23]. A study by Merli et al. did not find any significant difference in diastolic function according to the MELD status; however, LVDD occurred more in cirrhotic patients with ascites than those without ascites [27]. Somani et al. from India did not find any difference in echocardiographic parameters according to the degree of liver dysfunction (Child-Pugh class or MELD) [28]. However, the majority of previous studies had documented a significant relation of cardiac dysfunction with the severity of liver disease in cirrhotic patients, in accordance with the finding of our study.

Our study revealed that HRS was diagnosed in 15 patients (16%) during follow-up. The cirrhotic patients who developed HRS had significant LVDD as compared to those who did not develop HRS. A study by Arbol et al. revealed that diastolic dysfunction plays a key role in the impairment of effective arterial volume, leading to the development of type 1 hepatorenal syndrome in nonazotemic cirrhotic patients [24]. A study by Premkumar et al. from India found a significant correlation of degree of LVDD with liver function and complications of cirrhosis including renal dysfunction, sepsis, and hepatic encephalopathy, due to worsening of circulatory function [29]. Cirrhosis of the liver is associated with the reduction of effective arterial volume due to splanchnic arterial vasodilatation and reduced systemic vascular resistance and arterial blood pressure. In diastolic dysfunction, impaired cardiac chronotropic function due to reduced ratio of heart rate to noradrenaline can cause significant reduction of effective arterial blood volume, which can lead to decrease in renal perfusion and thus contribute to the pathogenesis of hepatorenal syndrome [24, 29, 30]. Moreover, cirrhotic patients with LVDD cannot increase the ventricular performance of the heart in response to stressful stimuli such as sepsis, leading to higher mortality [30, 31].

The previous studies have well documented the significance of the severity of LVDD on the outcome of liver transplantation or TIPS insertion in cirrhotic patients [16, 27, 32, 33]. However, the prognosis of the severity of LVDD on the survival of cirrhotic patients is not clearly defined in the previous literature. In our study, we found that the presence of LVDD as defined by *E*/*e*′ ratio ≥ 10 and low serum albumin were independent predictors of mortality in decompensated cirrhotic patients. A study by Karagiannakis et al. also found that the presence of LVDD and low serum albumin were significant predictor of mortality in patients with cirrhosis of liver in accordance with our findings [25]. Another study from Korea also documented that the presence of LVDD was an independent predictor of survival in multivariate analysis [10]. Anish et al. from Spain demonstrated that the *E*/*e*′ ratio is an independent predictor of survival in patients of cirrhosis of liver [23]. This important result of our study has a significant clinical implication, as impaired cardiac reserve due to diastolic dysfunction is masked many times by the presence of ascites; hence, the early diagnosis of diastolic dysfunction is necessary to improve the survival in decompensated cirrhotic patients.

The treatment of LV diastolic dysfunction in cirrhosis is aimed at facilitating myocardial relaxation and improving compliance of LV. However, no randomized controlled trials have evaluated the drugs for treatment of LVDD in cirrhosis of the liver. Beta blockers, angiotensin II receptor blockers, and aldosterone antagonists have been used to treat LVDD to control the heart rate and reduce cardiac overload [34]. A recent study by Premkumar et al. from India found that combination of ivabradine with carvedilol achieved a targeted heart rate of 55 to 65 beats per minute, so improves LVDD and survival in patients with cirrhosis [35]. Liver transplantation may be a possible treatment option for end stage liver disease patients with severe LVDD [10, 34]. Future clinical trials are aimed at improving LVDD in cirrhotic patients is needed to prolong survival in these patients.

As patients were included in the study regardless of etiology, all the previously alcoholic patients were thoroughly evaluated for the study. Those patients with abstinence of alcohol for more than 6 months were recruited in the study. Previous studies have shown a significant improvement of cardiac dysfunction after 6 months of alcohol abstinence [36]. Furthermore, according to our multivariate analysis, alcoholic etiology was not found to be an independent predictor of mortality as compared to nonalcoholic patients. Pozzi et al. from India and multiple western studies also found no difference in cardiac parameters between cirrhotic patients with alcohol and nonalcohol etiology [37, 38].

Our study has several limitations. A small number of cirrhotic patients (*n* = 92) were included from a single centre. Although the follow-up period was 24 months, longer follow-up time would have given more information on the prognostic role of LVDD in cirrhotic patients. Cardiopulmonary pressure measurements and neurohormonal measurements were not done in this study to assess cardiac chronotropic response to systemic circulatory dysfunction. The biochemical markers to support the diagnosis of cardiac dysfunction were not measured in our study. Effect of diuretics and infections like SBP on LVDD in decompensated cirrhotic patients were not assessed in our study. Despite these limitations, this study is promising. The prospective study design and real-life clinical data of decompensated cirrhotic patients were strengths of this study.

In conclusion, we demonstrated that the presence of LVDD as defined by *E*/*e*′ ≥ 10 and low serum albumin were independent predictors of mortality in decompensated cirrhotic patients. Therefore, cardiac evaluation by 2 D echocardiography with tissue Doppler imaging should be done in all decompensated cirrhotic patients. Early referral for liver transplantation and institution of specific medical therapy should be considered in patients having severe LV diastolic dysfunction to improve survival in patients with decompensated cirrhosis.

## Figures and Tables

**Table 1:** Demographic, clinical and echocardiographic data of decompensated cirrhotic patients enrolled in the study.

Parameters	Patients (*n* = 92)
Age in years	53 ± 9.85 (26-65)
Gender	Male 70 (76%) Female 22 (24%)
Etiology	Cryptogenic 45 (49%) Alcohol 30 (33%) HBV 13 (14%) HCV 2 (2%) Autoimmune 2 (2%)
Albumin (g/dl)	2.61 ± 0.68 (1.6-3.8)
Bilirubin (mg/dl)	2.43 ± 1.85 (0.9-13.5)
INR (s)	2.43 ± 1.85 (1-3.7)
Creatinine (mg/dl)	1.04 ± 0.63 (0.5-5.5)
MELD	16.73 ± 7.2 (7-36)
CTP	A 7 (7.6%) B 34 (36.9%) C 51 (55.5%)
Echocardiographic findings	LVEF 61.76 ± 8.67 (26-68) Mitral *E*/*A* ratio 1.14 ± 0.46 (0.68-3.04) *E*/*e*′9.46 ± 3.24 (6-18) Deceleration time 198.98 ± 32.33 (126-274) LAVI 28.74 ± 9.42 (19-49)

Abbreviations: LVDD: left ventricular diastolic dysfunction; HBV: hepatitis B virus; HCV: hepatitis C virus; INR: international normalized ratio; MELD: model for end-stage liver disease; LVEF: left ventricle ejection fraction; DT: deceleration time of E wave; *E*/*e*′ ratio, ratio of early diastolic annular velocity to peak early diastolic annular wave velocity; *E*/*A* ratio: ratio of early diastolic annular velocity to peak late diastolic arterial filling velocity; LAVI: left atrium volume index.

**Table 2:** Baseline clinical, echocardiographic data in decompensated cirrhosis patients, classified based on *E*/*e*′ ratio.

Parameters	*E*/*e*′ < 10 (*n* = 59)	*E*/*e*′ ≥ 10 (*n* = 33)	*p* value
Age in years	54.29 ± 10.24	51.40 ± 9.25	0.34
Hemoglobin (g/dl)	9.58 ± 1.01	9.48 ± 0.99	0.95
Albumin (g/dl)	2.91 ± 0.61	2.22 ± 0.5	0.01^∗^
Bilirubin (mg/dl)	2.16 ± 1.75	2.77 ± 1.94	0.009^∗^^#^
INR in sec	1.46 ± 0.42	1.99 ± 0.65	0.001^∗∗^^#^
Creatinine (mg/dl)	0.91 ± 0.35	1.04 ± 0.59	0.15^#^
Child-Pugh score	9.02 ± 1.74	10.25 ± 1.74	0.01^∗^
MELD	14.31 ± 5.83	19.88 ± 7.72	0.001^∗∗^
Sodium (mEq/l)	130.1 ± 7.38	131.67 ± 8.1	0.76
LVEF	61.48 ± 10.68	62.2 ± 5.1	0.68
*E*/*A* ratio	0.98 ± 0.32	1.34 ± 0.53	0.001^∗∗^
*E*/*e*′	6.99 ± 0.55	12.67 ± 2.35	0.001^∗∗^
DT	217.65 ± 26.64	174.7 ± 21.02	0.001^∗∗^
LAVI	24.08 ± 3.94	35.48 ± 6.73	0.001^∗∗^

Abbreviations: LVDD: left ventricular diastolic dysfunction; HBV: hepatitis B virus; HCV: hepatitis C virus; INR: international normalized ratio; MELD: model for end-stage liver disease; LVEF: left ventricle ejection fraction; DT: deceleration time of E wave; *E*/*e*′ ratio: ratio of early diastolic annular velocity to peak early diastolic annular wave velocity; *E*/*A* ratio: ratio of early diastolic annular velocity to peak late diastolic arterial filling velocity; LAVI: left atrium volume index; ^#^*p* value calculated by the Mann–Whitney *U* test.

**Table 3:** Echocardiographic parameters in decompensated cirrhotic patients, classified as per MELD status.

	MELD < 15 (*n* = 48)	MELD ≥ 15 (*n* = 44)	*p* value
LVEF (%)	60.83 ± 10.36	62.84 ± 5.93	0.68
Mitral *E*/*A* ratio	1.01 ± 0.45	1.31 ± 0.69	0.01
*E*/*e*′ ratio	8.74 ± 3.32	11.94 ± 4.24	0.001
DT (ms)	209.1 ± 33.2	185.45 ± 37.74	0.001
LAVI (ml/m^2^)	33.11 ± 8.51	26.58 ± 6.23	0.001
Diastolic dysfunction (DD)			
No DD	21 (43.7%)	11 (25%)	0.01
Grade 1 DD	16 (33.3%)	16 (36.3%)	
Grade 2 DD	11 (23%)	17 (38.6%)	

Abbreviations: LVEF: left ventricle ejection fraction; DT: deceleration time of E wave; *E*/*e*′ ratio: ratio of early diastolic annular velocity to peak early diastolic annular wave velocity; *E*/*A* ratio: ratio of early diastolic annular velocity to peak late diastolic arterial filling velocity; LAVI: left atrium volume index; LVDD: left ventricular diastolic dysfunction.

**Table 4:** Baseline characteristics of cirrhotic patients with moderate ascites who developed HRS and those who did not develop HRS during follow-up.

	HRS group (*n* = 15)	Non-HRS group (*n* = 48)	*p* value
Serum creatinine (mg/dl)	2.05 ± 1.05	0.82 ± 0.17	0.01
Serum sodium (mEq/l)	132.69 ± 7.47	134.54 ± 8.09	0.45
MELD score	24.92 ± 9.35	14.56 ± 5.43	0.002
Child-Pugh score	10.92 ± 1.49	9.38 ± 1.62	0.003
LVEF (%)	62.30 ± 7.05	61.34 ± 10.37	0.75
Mitral *E*/*A* ratio	1.30 ± 0.42	1.07 ± 0.34	0.04
*E*/*e*′ ratio	12.06 ± 3.22	8.86 ± 2.73	0.002
DT (ms)	185.08 ± 49.19	202.39 ± 32.42	0.16
LAVI (ml/m^2^)	34.21 ± 9.06	28.51 ± 7.51	0.02
Diastolic dysfunction (DD)			
No DD	1 (6.2%)	20 (26.3%)	0.03
Grade 1 DD	4 (25%)	26 (34.2%)	
Grade 2 DD	11 (67.7%)	30 (39.5%)	

Abbreviations: HRS: hepatorenal syndrome; MELD: model for end stage liver disease; LVEF: left ventricle ejection fraction; DT: deceleration time of E wave; *E*/*e*′ ratio: ratio of early diastolic annular velocity to peak early diastolic annular wave velocity; *E*/*A* ratio: ratio of early diastolic annular velocity to peak late diastolic arterial filling velocity; LVDD: left ventricular diastolic dysfunction.

**Table 5:** Cox regression model for predicting survival in patients with decompensated cirrhosis.

	Univariate analysis			Multivariate analysis		
	Hazard ratio	95% CI	*p* value	Hazard ratio	95% CI	*p* value
Age	0.97	0.97-1.03	0.17			
Male sex	0.6	0.18-1.98	0.4			
Hemoglobin (g/dl)	0.72	0.48-1.04	0.11			
Albumin (g/dl)	0.11	0.05-0.33	0.001	0.32	0.14-0.7	0.005
MELD	1.05	1.01-1.16	0.01			
Child-Pugh class	1.52	1.2-1.94	0.01			
Presence of ascites	2.02	1.14-4.16	0.05	1.33	0.59-2.96	0.48
Alcohol vs. other etiology	1.72	0.8-3.6	0.72			
LVEF (%)	1.02	0.96-1.08	0.27			
*E*/*e*′ ≥ 10 presence of LVDD	4.19	1.78-9.88	0.001	2.72	1.07-6.9	0.03
Alcohol vs. other etiology	1.72	0.8-3.6	0.72			

Abbreviations: LVDD: left ventricular diastolic dysfunction; MELD: model for end-stage liver disease; LVEF: left ventricle ejection fraction; *E*/*e*′ ratio: ratio of early diastolic annular velocity to peak early diastolic annular wave velocity.

## Data Availability

Data is available on request.
